# Seasonal mass vaccination with R21/Matrix-M for malaria elimination (SERVAL): protocol of the cluster randomised trial

**DOI:** 10.1186/s13063-025-09048-6

**Published:** 2025-09-29

**Authors:** Edgard D. Dabira, H. Magloire Natama, Fatou Jaiteh, Koen Peeters Grietens, Fadima Y. Bocoum, Mamadou Ousmane Ndiath, Nuredin Mohammed, Balla Gibba, Adrian V S Hill, Azra Ghani, Annette Erhart, Halidou Tinto, Umberto D’Alessandro

**Affiliations:** 1 Disease Control & Elimination Theme, Medical Research Council The Gambia Unit at London School of Hygiene and Tropical Medicine (MRCG at LSHTM), Fajara, The Gambia; 2 Unité de Recherche Clinique de Nanoro, Institut de Recherche en Sciences de La Santé- Direction Régionale du Centre Ouest (URCN/IRSS-DRCO), Nanoro, Burkina Faso; 3 Department of Public Health, Institute of Tropical Medicine, Socio-Ecological Health Research Unit, Antwerp, Belgium; 4 Institut de Recherche en Sciences de La Santé (IRSS), Ouagadougou, Burkina Faso; 5 National Malaria Control Programme, Banjul, The Gambia; 6 Centre for Clinical Vaccinology and Tropical Medicine, The Jenner Institute, University of Oxford, Oxford, UK; 7 MRC Centre for Global Infectious Disease Analysis, Department of Infectious Disease Epidemiology, Imperial College London, London, UK; 8 Saw Swee Hock School of Public Health, National University of Singapore and National University Health System, Singapore, Singapore; 9 Lee Kong Chian School of Medicine, Nanyang Technological University, Singapore, Singapore

**Keywords:** R21/Matrix-M, Seasonal mass vaccination, Malaria, Cluster randomised trial, The Gambia, Burkina Faso

## Abstract

**Introduction:**

Progress in malaria control has stalled since 2015, highlighting the need for new control tools. The R21/Matrix-M (R21) malaria vaccine, a pre-erythrocytic vaccine recently approved by WHO for small children, may be one of these tools. This trial aims to assess whether seasonal mass vaccination with R21 reduces malaria transmission in The Gambia and Burkina Faso, two countries at the extreme of the transmission spectrum.

**Methods:**

This is a multi-centre open cluster-randomised controlled trial to assess the impact of mass vaccination with R21 on malaria transmission and morbidity. The trial will be implemented in eastern Gambia (low to moderate transmission) and Central Burkina Faso (intense transmission). Thirty medium-sized villages in The Gambia and 24 in Burkina Faso will be randomised (1:1) to either intervention or control arm. All eligible residents in intervention villages will receive R21 vaccinations in three-monthly rounds, from May to July 2024, prior to the malaria transmission season. A booster vaccine dose will be administered the following year, in June 2025. The primary outcome is malaria prevalence at peak transmission (November 2024). Secondary outcomes include safety and tolerability, incidence of clinical malaria, vaccination coverage and community acceptability, cost and cost-effectiveness of the intervention.

**Discussion:**

This is the first trial on seasonal mass vaccination aiming at reducing malaria transmission. Strengths of the study include its design as an adequately powered cluster-randomised trial and the inclusion of study sites with differing transmission intensity which will also provide safety and efficacy data for different age groups. Key challenges remain vaccine hesitancy and vaccination coverage. If successful, R21 seasonal mass vaccination will be an innovative intervention to accelerate malaria elimination efforts and reach the goal set in the Global Technical Strategy for malaria 2016–2030.

**Trial registration:**

Clinical trials.gov, NCT06578572. Registered on 27 March 2024.

## Background

Since 2000, the global burden of malaria has significantly reduced thanks to the large-scale implementation of control interventions. Between 2000 and 2023, an estimated 2.2 billion malaria cases and 12.7 million malaria deaths were averted worldwide, with 1.7 billion cases and 12 million deaths prevented in the World Health Organization (WHO) African Region alone [1]. During the same period, 26 countries successfully reported 3 consecutive years of zero indigenous malaria cases, and 18 of them were certified malaria-free by WHO [1]. Nevertheless, malaria remains a major public health problem, particularly in sub-Saharan Africa where progress has stalled since 2015 [2]. In 2023, there were an estimated 263 million malaria cases and 597,000 deaths, most of them in Africa [1].

Malaria affects disproportionately people experiencing disadvantage, poverty, and exclusion. It is both a consequence and a driver of poverty and inequality [3, 4]. A malaria-free world, besides preventing disease and deaths, would stimulate development and economic growth, improving the life of hundreds of millions of people. Nevertheless, achieving malaria elimination and possibly eradication would require new tools, innovative interventions and enhanced local capacities for the implementation of increasingly complex control measures. A vaccine against malaria could be one of these new tools.

Malaria vaccine development has so far targeted infants as malaria mortality is the highest in children under 5. However, mathematical models suggest that mass vaccination with a pre-erythrocytic vaccine (PEV) may substantially reduce population-level malaria transmission [5]. Two PEVs have been recently approved for use in small children by the WHO, namely RTS,S/AS01 (RTS,S) in October 2021 and R21/Matrix-M (R21) in October 2023 [6]. In Ghana, Malawi and Kenya, RTS,S, introduced by the national immunisation programmes in 2019 through large-scale pilot schemes, decreased hospital admissions for severe malaria by 32%, and all-cause mortality by 9% [7]. R21 is similar to RTS,S as it includes hepatitis B surface antigen (HBsAg) fused to the C-terminus and central repeats of the circumsporozoite protein (CSP), although it lacks the excess of HBsAg found in RTS,S. A double-blind, randomised, phase 3 trial across five sites in four African countries with differing malaria transmission intensities and seasonality reported in children aged 5–36 months a vaccine efficacy against multiple episodes of clinical malaria between 67 and 75% [8]. Both vaccines are safe and well tolerated [7–9].

Besides children < 5 years, older age groups are also at risk of malaria even though the risk of severe disease is lower than in younger age groups [10, 11]. In a context of decreasing but residual transmission, malaria can be an important cause of death among young adults [12]. Moreover, given R21 is a PEV, it protects against infection and may have a substantial effect on malaria transmission when administered to the whole population. We propose to carry out a cluster-randomised trial to determine the impact of seasonal R21 mass vaccination (all ages) on malaria transmission and morbidity. The trial will be implemented in 2 sites at the extreme of the transmission spectrum, i.e. in The Gambia where malaria transmission is moderate-to-low and in Burkina Faso where it is intense.

### Trial objectives

The primary objective of the trial is to compare in intervention and control clusters the prevalence (all age groups) of malaria at peak transmission after seasonal mass vaccination with R21 (3 monthly doses). Secondary objectives are to [1] assess the safety and tolerability of R21; [2] compare in intervention and control clusters the incidence of malaria infection and clinical malaria following the 3-monthly rounds of seasonal mass vaccination (primo-vaccination) and after one booster dose; [3] compare the malaria prevalence in both arms at the peak transmission after the 1-year booster dose; 4) determine the coverage of the intervention and the related socio-cultural factors; and [5] assess the community acceptability and cost-effectiveness of the intervention.

## Methods

This study protocol has been developed according to SPIRIT (Standard Protocol Items: Recommendations for Intervention Trials) guidelines [13].

### Study design and settings

This is a multi-centre open cluster-randomised controlled trial (ClinicalTrials.gov, NCT06578572) to assess the impact of mass vaccination with R21 on malaria transmission and morbidity. The trial follows a superiority framework, aiming to evaluate whether mass vaccination with the R21 malaria vaccine would result in a statistically significant reduction in malaria transmission and morbidity compared to no mass vaccination (control arm). The trial employs a parallel group design within a cluster-randomised framework, in which distinct clusters (e.g. villages) are randomly assigned to either the intervention arm (receiving mass vaccination) or the control arm (no vaccination). These groups are followed over time to assess differences in outcomes, allowing for a comparison of the vaccine’s effectiveness at the community level.

The study clusters are medium-sized villages with a population of 200–600 inhabitants. Study villages will be randomised in a 1:1 ratio to either the intervention or the control arm, and mass vaccination with R21 will be implemented in the intervention villages. All study villages, regardless of the study arm, will receive standard malaria control interventions as implemented by the National Programmes, and according to the National Strategic Plan for malaria control. These are Seasonal Malaria Chemoprevention (SMC), Intermittent Preventive Treatment during pregnancy (IPTp), Insecticide-Treated bed Nets (ITNs) and prompt diagnosis and treatment with an effective antimalarial drug.

The study will be implemented in Upper River Region (URR) in the eastern part of The Gambia and in Boussé District in the Central region of Burkina Faso. The URR is a region characterised by a long dry season from mid-October to mid-June, followed by a single short rainy season. Malaria transmission is moderate to low, and highly seasonal, with most malaria cases occurring during the rainy season and immediately afterwards, until December–January [14]. In November 2019, malaria prevalence by molecular methods was 12.8%, while the incidence rate of clinical malaria was 1.1/100 person-months [15]. In the Central region of Burkina Faso, malaria transmission is intense and seasonal. In 2020, a total of 103,314 cases of uncomplicated malaria and 2937 cases of severe malaria were reported. Most of them occurred over 5 months of the year, from July to November [16]. In both countries, malaria is predominantly due to *Plasmodium falciparum*, the main vector is *Anopheles gambiae* and the first-line treatment is artemether-lumefantrine.

### Village selection and Informed consent

The year preceding the implementation of mass vaccination, a baseline cross-sectional survey will be conducted in November, at the peak of malaria transmission, to identify villages with the required malaria prevalence. The survey will be implemented in 45 Gambian villages in the URR and 30 Burkinabe villages in Boussé District. In The Gambia, 30 villages (clusters) with a baseline *Plasmodium falciparum* prevalence by qPCR of at least 10% will be selected and included in the study; for Burkina Faso, 24 villages with a baseline malaria prevalence of at least 30% will be selected and included in the study. In each country, selected villages will be randomised to either intervention or control arm in a 1:1 ratio using a computer-generated algorithm (R version 4.2.2 and STATA version 16). Randomisation will be constrained such that the mean baseline prevalence in the intervention clusters is within ± 10% of the prevalence in the control clusters.

Following a thorough explanation of the study objectives and methods to the local authorities and the population of the study villages through sensitisation meetings, a census will be conducted to determine the size of the population, age structure and use of standard malaria control interventions. Afterwards, the study team, including field workers and nurses, will initiate individual consent procedures (before any study-related activity is implemented) to obtain written informed consent from all willing residents. Parents/guardians will provide written informed consent for their children, while adolescents 12–17 years old will be asked to provide an additional written assent. The consenting process will continue during the trial to include new residents and those missed at the time of the initial consenting process. Each resident will be assigned a unique census identification number.

### Study population and eligibility criteria

The target population for the R21 mass vaccination campaign are all eligible residents in the intervention villages. Residents in intervention villages will be enrolled for vaccination according to well-defined inclusion criteria as follows: [1] age ≥ 5 months, [2] willingness to comply with trial procedures, and [3] individual written informed consent. Exclusion criteria include the following: [1] pregnancy, [2] history of allergic disease or reactions likely to be exacerbated by any component of the vaccines, [3] any history of anaphylaxis in relation to vaccination, [4] known chronic illness, [5] any other significant disease, disorder or situation which, in the opinion of the investigator, may either put the participants at risk because of participation in the trial, or influence the result of the trial, or the participant’s ability to participate in the trial. To be vaccinated, all inclusion criteria and none of the exclusion criteria should be met. Prior to each vaccine dose, women of reproductive age (15 to 49 years old) will be systematically tested for pregnancy. Moreover, eligibility will be checked for all study participants during each vaccination round, and before the administration of the vaccine dose.

## Intervention

### Study vaccine: R21/Matrix-M

The R21 vaccine is made up of R21 and the adjuvant Matrix M. R21 is a recombinant protein vaccine that contains a component of the circumsporozoite protein (CSP) *of Plasmodium falciparum* fused to hepatitis B surface antigen in a form of non-infectious virus-like particle produced in yeast cell (Hansenula) [8]. R21 is manufactured by the Serum Institute of India (SIIPL). Matrix M, developed by Novavax, is a highly immunogenic saponin-based adjuvant that stimulates both humoral and cellular immune responses to vaccines.

Two vaccine doses will be available: a 5 µg of R21 adjuvanted with 50 µg of Matrix M (Recombinant, Adjuvanted) and a 10 µg of R21 mixed prior to administration with 50 µg of Matrix M (Recombinant). The 5 µg dose is formulated as a single vial of 1.24 ml, pre-packed for intramuscular injection for 2 eligible study participants aged < 15 years. The 10 µg dose is obtained by mixing prior to administration 2 vials of R21 (0.65 ml/vial) and 1 vial of Matrix M (0.5 ml). The mixing consists of withdrawing 0.5 ml from each vial of the R21 and adding them to the vial of 0.5 ml of Matrix M to obtain a mixture of 1.5 ml, which is gently mixed and then 0.75 ml (corresponding to a vaccine dose of 10 µg) is withdrawn and administered intramuscularly to eligible study participants aged ≥ 15 years.

### Mass vaccination

**Fig. 1:**
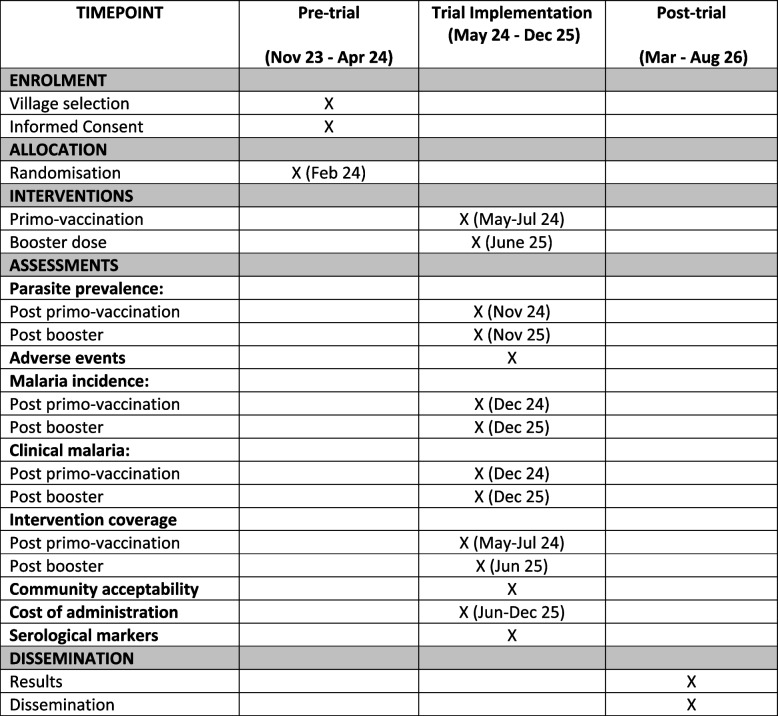
Trial timelines (SPIRIT figure). “*X*” indicates when the activity occurs

The study intervention*, *i.e., mass vaccination with R21, will be implemented over 2 years in 2024 and 2025, covering two malaria transmission seasons. Three monthly doses of R21 (primo-vaccination) will be administered to all eligible residents in intervention villages, starting from May 2024, with the aim of completing the vaccination schedule by July 2024, before the start of the malaria transmission season. A booster vaccine dose will be administered the following year, in June 2025, to all eligible individuals who received at least one vaccine dose the previous year. Residents who are eligible but not vaccinated in the previous year will be offered a complete vaccination schedule, i.e. 3 monthly doses, starting from April 2025 (Fig. 1).

### Post-vaccination activities

In each village, during each series of vaccination, the first 100 vaccinated individuals will be actively visited at home daily for 3 consecutive days, and then at day 7 after the vaccination to collect local and systemic adverse events (AEs). All vaccinated individuals (or their parents for those < 18 years) will be requested to report to the study nurse stationed in their study village in case of any complaints or symptoms occurring within 28 days post-vaccination.

In each intervention village, 10 randomly selected individuals (≥ 3 years old) will have 1.5 ml of venous blood collected at 4 time points, i.e. prior to each monthly dose and 1 month after the third vaccine dose, for safety laboratory (haematology and biochemistry) tests.

Following primo-vaccination, a system of passive case detection will be established at local health facilities and within each study village where a study staff (nurse or fieldworker) will be stationed permanently throughout the study duration to record clinical malaria cases. All suspected malaria cases (patients with fever and/or history of fever without any other obvious illness) will be investigated with a malaria Rapid Diagnostic Test (RDT) and treated following the national guidelines. Axillary body temperature, a thick blood film for microscopy, and dried blood spots (DBS) on filter paper for later molecular analysis will be collected.

To estimate the incidence of malaria infection, 20 individuals per village in Burkina Faso and 30 individuals per village in The Gambia aged at least 6 months will be randomly selected and visited monthly at home after the completion of the primo-vaccination schedule. Before starting the monthly follow-up, they will be treated with a full dose of artemether-lumefantrine to clear any parasitaemia. During the home visits, the axillary body temperature will be measured and a finger prick blood sample for a thick blood film for later microscopy and DBS for later molecular analysis will be systematically collected until the end of the transmission season (December 2024). Similarly, during the dry season, three additional home visits will be conducted every 8 weeks ± 2 weeks between January and May 2025 and during the same period in 2026.

In all study villages, a cross-sectional survey to estimate the prevalence of malaria infection (all ages) will be carried out at peak transmission season (November) both after the primo-vaccination (Nov. 2024) and after the booster dose (Nov. 2025). One hundred individuals per village aged at least 6 months will be randomly selected, regardless of their vaccination status. All surveyed participants will be clinically examined, including measurement of the axillary temperature, and a finger prick blood sample will be collected for a thick blood film for later microscopy and DBS for later molecular analysis. Individuals with symptoms suggestive of clinical malaria will have a standard RDT performed on them. If positive, they will be treated with artemether-lumefantrine. A short questionnaire will be administered to collect information on recent travel history, history of clinical malaria, and use of preventive measures against malaria.

### Anthropology, Health economics and Mathematical modelling

Prior to starting the trial and during the intervention, socio-cultural factors in relation to the intervention’s effectiveness, including its local acceptability, will be assessed using ethnographic qualitative methods (participant observation, in-depth interviews and group discussions). Before the mass vaccination campaign, potential bottlenecks for the intervention will be assessed, and recommendations will be made to improve its implementation. This will help fine-tune the implementation of the intervention to local realities in addition to engaging stakeholders to ensure participation and retention. During this phase, we will involve community members and stakeholders in the process of developing/adapting the mass vaccination implementation strategy to increase coverage of the intervention. A comprehensive stakeholder assessment will be carried out at the beginning of the project, aiming to identify formal and informal groups and organisations active in the communities.

A health economics study on the cost-effectiveness of the intervention will be carried out. A cross-sectional survey will be carried out at the beginning of the project in both study arms to estimate the cost of seeking care among households and the costs supported by the health facility. Cost of mass vaccination will be collected for each round. Questionnaires will be administered to collect all the data at household and health facility levels.

Mathematical modelling will be used to generalise the findings from the trial to provide direct support for policy decision-making. This will build on extensions of an existing modelling framework that has been used to support decision-making for the RTS,S vaccine [17, 18].

### Study outcomes

The primary endpoint is the prevalence of malaria infection measured by molecular methods (qPCR) in all age groups as determined by a cross-sectional survey at the peak of the first transmission season, i.e. in November 2024, following the primo-vaccination. Secondary endpoints are as follows: [1] occurrence of AEs; [2] incidence of malaria infection in all age groups during the transmission season following primo-vaccination; [3] incidence of clinical malaria in all age groups following primo-vaccination; [4] prevalence of malaria infection by molecular methods in all age groups at peak transmission following the booster dose; [5] incidence of malaria infection in all age groups during the transmission season following the booster dose; [6] incidence of clinical malaria in all age groups following the booster dose; [7] coverage of the intervention and community acceptability; [8] cost of vaccine administration, both for the primo-vaccination and the booster dose; [9] incremental cost-effectiveness. Exploratory outcomes are prevalence of serological markers (AMA-1, MSP-1, GLURP, GexP, RH2030 and Etramp5.Ag1) at peak transmission after the mass vaccination campaign with 3 monthly doses and the booster dose.

### Sample size and statistical analysis

**Fig. 2:**
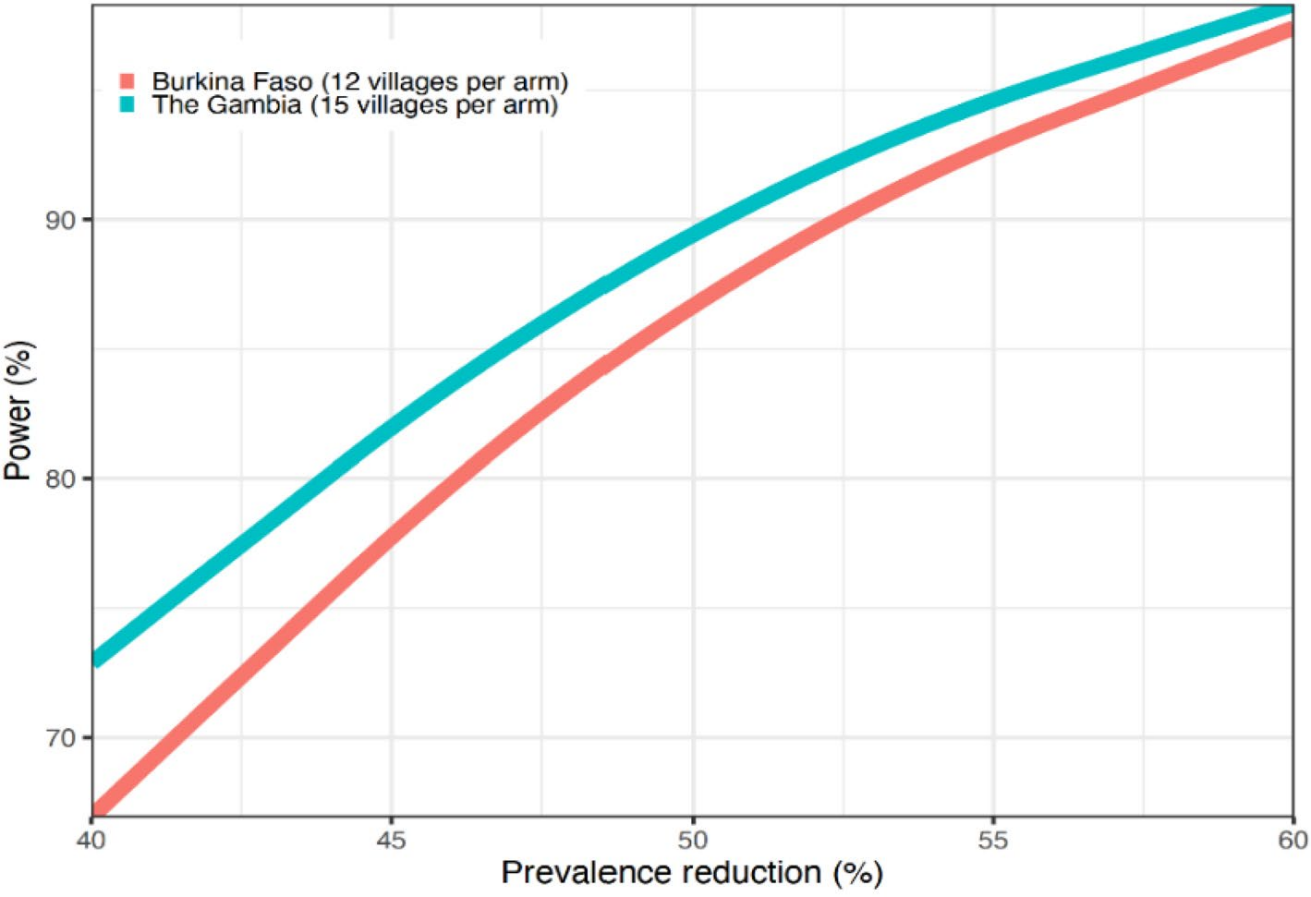
Power by prevalence reduction by country (Burkina Faso and The Gambia)

The primary analysis will be a comparison of the cluster level malaria prevalence rates following the primo-vaccination (3 doses) between the two arms. Arms will initially be compared by a two-samples one-sided *t*-test (2.5%), and, to be conservative, the variance between prevalence in each arm will be assumed unequal (Welch approximation). The intra-cluster correlation coefficient (ICC) is estimated at 0.17 for Burkina Faso [19] and at 0.10 for The Gambia [15]. The malaria prevalence in the control arm is assumed to be 40% in Burkina Faso and 13.5% in The Gambia [15]. Assuming malaria prevalence in the intervention arm will decrease by about 45% compared to the control arm, 12 cluster per arm in Burkina Faso and 15 cluster per arm in The Gambia with 100 individuals per cluster would be needed to detect such a reduction between study arms with 80% power and a 5% significance level. The sample size has been estimated for each country separately as the impact of the intervention may differ between countries (Fig. 2).

**Fig. 3:**
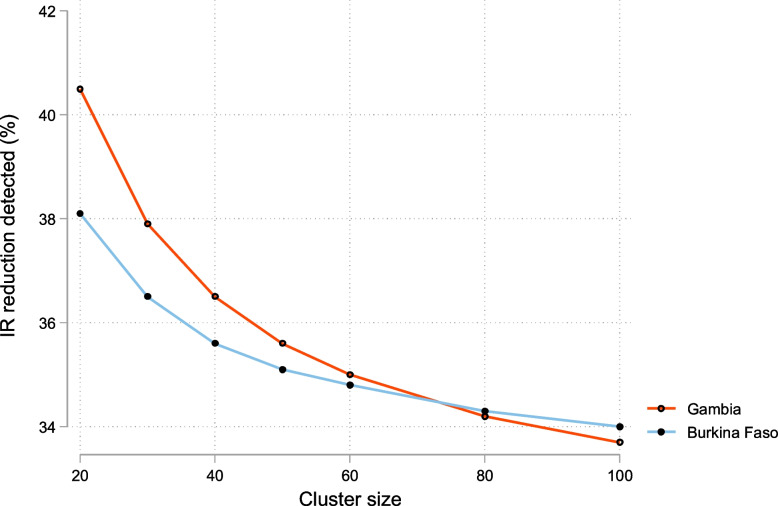
Reduction of incidence rate detected by sample size at 80% power

As for the incidence of infection, this will be determined by collecting monthly blood samples from randomly selected individuals. In The Gambia, in 2013–2014, the incidence of infection in the study area was 1.42 per person-year [14]. In Burkina Faso, the incidence of infection is assumed to be at least twofold higher, i.e. 2.8 per person-year. Applying the same assumptions for the primary endpoint (e.g. ICC), 20 individuals per village in Burkina Faso and 30 in The Gambia will be able to detect a 38% reduction in the incidence of infections at 80% power. This estimation considers the number of clusters both in Burkina Faso and The Gambia (Fig. 3).

The primary outcome, prevalence of malaria infection, defined as the proportion of sampled residents carrying the parasite (*P. falciparum*) detectable by varATS PCR, will be compared between study arms using random effects logistic regression (with random effect on village) adjusting for study month, age group (< 5, 5–15 and > 15), ITN use and baseline malaria prevalence by village.

For secondary outcomes, incidence of clinical malaria will be estimated by recording all clinical cases from study villages (both intervention and control) attending the local health facilities or diagnosed by study nurses based in study villages (passive case detection). Incidence will be compared between arms using random effects Poisson regression—a random effect for study village will be included for the clustering. Incidence of malaria infection will be estimated by the number of first or only infections over person-months of follow-up. Intervention coverage will be measured by dividing the number of individuals having completed the vaccination schedule (3 monthly doses) by the number of individuals eligible for vaccination. In addition, coverage of at least one vaccine dose will be estimated. Coverage will be calculated for each vaccination round and intervention year, while community acceptability will be assessed qualitatively in both the intervention and control arms. Vaccination costs and cost-effectiveness will be estimated by the annual costs of the vaccination campaign and the vaccine delivery cost per vaccinated individual. The cost-effectiveness will be estimated for the intervention (mass vaccination campaign) compared to the status quo (no mass vaccination campaign).

The exploratory outcome, the prevalence of serological markers (AMA-1, MSP-1, GLURP, GexP, RH2030 and Etramp5.Ag1), will be compared between arms using random effects logistic regression (with random effect on village).

Missing data percentages will be reported; available case analysis will be performed unless there is a higher missing data rate (> 15%) in which case we will consider using multiple imputation for the analyses. Primary analysis will be based on intention-to-treat approach; however, we will explore a treatment effect separately for those receiving the full dose as per the protocol and those receiving partial doses in sensitivity analyses.

#### Randomisation and blinding

Randomisation will be stratified by country. The unit of randomisation will be the village (cluster) with about 200 to 600 inhabitants. Allocation of each of the selected villages to one of the trial’s arms (intervention/control) will be done through computer-based randomisation by the study’s statistician. To prevent imbalance for potential confounding factors, restricted randomisation to balance the arms on factors such as baseline prevalence, ITN coverage, and distance from health facilities will be used [20].

In this cluster-randomised trial, blinding residents to vaccination (*i.e.,* placebos) would not be justified considering the logistical challenges and costs, and the likely limited impact on outcome measures. Nevertheless, all laboratory staff involved in processing samples and evaluations contributing to the study outcomes will be blinded to the allocation arm (intervention or control arm).

### Laboratory processes

A blood sample (finger prick) will be collected from all selected individuals. Part of it will be used to perform an RDT when applicable, and a thick blood film; the rest (a few droplets) will be blotted onto Whatman 3MM filter paper (three blood spots). Filter papers will be air dried and stored with desiccant at 4 °C for up to one week and then stored at − 80 °C for later use. Laboratory evaluations will include microscopy, haematology and biochemistry safety laboratory evaluation, molecular detection of *P. falciparum* parasitemia and serological analyses.

### Microscopy

Thick films will be stained with 10% Giemsa and examined under 1000-fold magnification by trained, experienced microscopists. A slide will be declared negative after reading 200 high-power fields. Once detected, parasite density will be estimated by counting the number of asexual parasites per white blood cell (WBC) until 500 WBCs have been counted and assuming a mean WBC count of 8000/μl. Quality control will be performed on 10% of randomly selected slides.

### Hematology and biochemistry analyses

Clinical laboratory tests (safety laboratory), haematology and biochemistry (Alanine Aminotransferase (AST), Alanine Aminotransferase (ALT), Urea, Creatinine) will be done. Haematology testing will be done using Sysmex XN-350, while biochemistry testing will be done using Cobas c 111. Safety laboratory values after vaccination will be compared with baseline data to identify any clinically significant deviations from baseline.

### Molecular detection of P. falciparum parasitemia

DNA will be extracted from filter papers by Qiagen extraction method using automated mid-to-high throughput nucleic acid purification (QIAcube HT) robot. Two 6 mm punches of one of the spots on the filter paper, each representing approximately 8 µL of blood, are added to a 96-deep-well plate and used for DNA extraction with established protocols at MRCG Fajara. The extracted DNA will be temporarily stored at 4 °C before varATS qPCR. Long-term storage will be at − 80 °C. Extracted DNA will be analysed in duplicate with varATS qPCR using 5 µL of extracted DNA per assay [21]. All qPCR output will be analysed using the BioRad CFX Manager software. Each plate contains two rows of the 3D7 standard dilution, which will be used for the quantification curve and allow quality control. The limit of detection is 0.01 p/µl. Samples above the limit of detection (LOD) with a normal curve will be scored as positive, and those below the limit of detection will be scored as negative.

### Serological analysis

The multiplex bead-based platform (MAGPIX© qSAT) will be utilised for serological analysis. This platform uses different fluorescent beads to measure simultaneously up to 200 different targets, including both antigens and antibodies, with as little as 10 µL of DBS.

Antibodies will be eluted from DBS (6 mm punches) which have been collected on Whatman filter paper for each participant. A bead-based multiplex assay for measuring humoral responses to multiple malaria antigens in a single sample (multiplexing) will be used to measure IgG antibody responses (median fluorescence intensity and seropositivity rates). Species-specific and non-cross-reactive antigens such as MSP1-19, CSP, LSA, AMA1, GLURP, PfEMP1-PF13, PfGLURP, PfSALSA, PfLSA3, PfLSA1-41 and gSG6 (Saliv1) will be used to measure antibody responses to *Plasmodium *spp. Each protein will be covalently coupled at a concentration of 4 μg Ag/106 beads (MagPlex microspheres, Luminex Corp., Austin, TX, USA). Luminex beads will be provided by collaborators at the London School of Hygiene and Tropical Medicine (LSHTM) and serology using the multiplex platform will be performed according to established protocols at the Medical Research Council Unit The Gambia (MRCG). As controls for the immunoassay, BSA-coupled beads, a pool of negative sera (non-exposed European sera) and positive sera (highly exposed Gambian sera from previous surveys) will be added to each plate. Plates will be analysed and read using the MAGPIX® system with a minimum amount of 400 beads per spectral address. Results will be presented as the median fluorescent intensity.

### Data collection and management

To ensure the trial procedures are implemented consistently across all sites, several workshops and training sessions will be organised for the research team in both The Gambia and Burkina Faso. Additionally, standard operating procedures and standardised research data collection tools (questionnaires and forms) will be developed. Quality control of the data will be done routinely during field supervision visits.

Data for each participant will be captured on electronic case report forms (eCRFs). Informed consent status, demography, medical history, physical examination, vital signs, vaccine administration, adherence to vaccination schedule, adverse events, and laboratory evaluations will be recorded. All trial data will be stored and managed within the REDCap trial database system (Software – REDCap), an application specifically designed to collect and store clinical trial data and customised for electronic data capture at the trial site.

### Trial oversight and monitoring

The study Principal Investigators are primarily responsible for overseeing and auditing the conduct of the clinical trial. Principal Investigators and study personnel in each participating country will engage in regular coordination meetings throughout the vaccination campaigns and data collection phases. Continuous data review will be undertaken by the study team to ensure completeness, accuracy, and consistency. Study progress will be evaluated and discussed through regular meetings, which will include the study’s Monitors. Any unforeseen events or protocol deviations encountered during the study will be systematically documented and promptly reported to the appropriate ethics committee and regulatory body. In addition, the quality of data and adherence to Good Clinical Practice (GCP) standards will be monitored by independent and experienced GCP monitors.

### Safety management

An independent data and safety monitoring board (DSMB) will be appointed to advise the sponsor and investigators on the trial’s safety issues. Prior to the implementation of the trial, study staff (nurses and physicians) will be trained in adverse events assessment and reporting. All serious adverse events will be reported to the local ethics committee and the DSMB.

A study participant will be discontinued from participation in the study if any clinically significant adverse event (AE), laboratory abnormality, intercurrent illness or other medical condition or situation occurs such that continued participation in the study would not be in the best interest of the participant.

### Confidentiality of trial data and result dissemination

Information on study participants will remain confidential. Unique identifiers will be used on case report forms (CRFs) and samples. CRFs will be kept in locked files; and electronic data on tablets will have password securities accessible only to authorised study team members. All identifiable information will be delinked from data before being transferred to the study database.

In each country, the results of the trial will be presented to the Ministry of Health through the National Control Programme and local stakeholders. Results will also be presented at international conferences and published in peer-reviewed journals*.*

## Discussion

The SERVAL trial aims to determine the impact of seasonal mass vaccination with R21 on malaria burden, i.e. prevalence of infection, incidence of clinical malaria and malaria infections. This is the first time the potential effect of vaccinating the entire population with a vaccine developed for children under 5 years of age is being investigated. While the R21 vaccine was first tested in adults before progressing to phase II and III trials in children [22], there remains limited data on its efficacy in protecting older children and adults from infection. The administration of R21 vaccines to healthy volunteers both in the United Kingdom (UK) and Burkina Faso was safe and elicited similar IgG immune responses against the conserved central NANP repeat region of the pre-erythrocytic CSP [23]. Furthermore, in adult volunteers who received the standard regimen of three doses of R21 at four-week intervals and were then experimentally infected with malaria, the vaccine demonstrated an efficacy of 80% [22]. Mathematical models suggest that mass vaccination with a pre-erythrocytic vaccine may substantially reduce population-level malaria transmission [5]. Mass vaccination before the transmission season at a coverage between 60 and 80% may reduce malaria prevalence by 70–80%, while combining it with mass drug administration would almost interrupt transmission [24]. It is important to confirm or refute such predictions through large-scale trials especially given that some goals outlined in the WHO Global Technical Strategy 2016–2030 are lagging by over 60% [25]. Urgent new control tools and strategies are needed to regain progress towards malaria elimination and eventual eradication. The mass vaccination of the entire population with R21 could be one such approach.

Cluster-randomised trials are the gold standard to assess the community-level effect of an intervention [20, 26]. Key considerations for the design of cluster-randomised trials are the number of clusters and their sizes [26, 27]. The SERVAL trial is a multicentre one, implemented in 2 countries with differing malaria transmission, i.e. The Gambia where malaria transmission is moderate to low while in Burkina Faso it is intense. The inclusion of both countries relates to the protective efficacy of R21, whose impact may vary with the intensity of transmission. Implementing the trial in two countries with extreme transmission rates will ensure the applicability of results to most African countries with seasonal malaria transmission.

Vaccine hesitancy is a significant challenge for mass vaccination efforts and is defined as delays in acceptance or refusal of safe vaccines despite the availability of vaccination services [28]. This complex phenomenon is influenced by factors such as mistrust, misinformation, cultural beliefs and personal experiences which negatively impact vaccine uptake and vaccination coverage. Addressing vaccine hesitancy requires effective community engagement strategies that build trust, provide accurate information, and encourage open dialogue. Engaging communities to identify the most effective, ethical, and sustainable ways to implement mass vaccination is crucial for achieving high coverage. Therefore, an ethnographic approach will be used to explore vaccine hesitancy and inform the trial’s implementation.

Estimating vaccination coverage in mass campaigns is challenging due to various logistical and social factors, including population movements. A qualitative study conducted in the URR in The Gambia showed that the main barriers to participation and adherence in mass drug administration campaigns were the long- and short-term mobility of individuals and specific subgroups [29]. Mobile populations may not be fully accounted for, leading to inaccurate estimates of true coverage. Nonetheless, with extensive community engagement and regular updates of study population census as planned in the trial, it is hoped that participant retention will be optimal, and coverage estimates accurate.

With limited resources, along with a growing gap between available funding and resources needed to meet the WHO’s Global Technical Strategy (GTS) targets, it has become increasingly important to evaluate the cost and the cost-effectiveness of new interventions to ensure that scarce funding is allocated efficiently. Therefore, we will also estimate the cost and cost-effectiveness of seasonal mass vaccination with R21 in each study site.

This is the first cluster randomised trial on seasonal mass vaccination to reduce malaria transmission. Strengths of the study include its design as an adequately powered cluster randomised trial and the inclusion of study sites with differing transmission intensity which will also provide safety and efficacy data for different age groups, i.e. older children and adults, that were not included in the large phase II and III trials implemented to register the vaccine. If mass vaccination with R21 shows a major impact on malaria transmission, it will become a powerful tool to further reduce malaria burden. However, as previously mentioned, potential challenges of mass vaccination include vaccine hesitancy and population movements, which may reduce vaccine coverage to levels where no or little effect will be achieved.

## Conclusion

This cluster randomised trial explores the impact of seasonal mass vaccination with R21 as an innovative intervention to accelerate the interruption of malaria transmission and to reduce the malaria burden. If successful, R21 mass vaccination has the potential to save lives, improve economic stability, and bring communities closer to a malaria-free future.

Trial status.

The trial is ongoing, and the current protocol is version 4.0 (20 September 2024). Field activities for the first year of mass vaccination (2024) are conducted between May and December 2024. The second year activities (2025) for the booster dose administration will be implemented between April and December 2025. Trial results are expected to be available in March 2026.

## Data Availability

Trial data and documents will be stored in the Medical Research Council Unit The Gambia’s archives and made available on request.

## Abbreviations

CSP: Circumsporozoite protein
GTS: Global Technical Strategy
HBsAg: Hepatitis B surface antigen
ICC: Intra-cluster correlation coefficient
ITNs: Insecticide-treated bed Nets
R21: R21/Matrix-M
AEs: Adverse events
ALT: Alanine aminotransferase
AST: Aspartate aminotransferase
CRFs: Case report forms
DBS: Dried blood spots
DSMB: Data and Safety Monitoring Board
GCP: Good Clinical Practice
IPTp: Intermittent Preventive Treatment during pregnancy
PCD: Passive case detection
qPCR: Quantitative polymerase chain reaction
RDT: Rapid Diagnostic Test
SMC: Seasonal Malaria Chemoprevention
WHO: World Health Organization
